# Integrative taxonomy reveals two new species of *Cortinarius* section *Telamonia* (*Cortinariaceae*, *Agaricales*) from southwestern China

**DOI:** 10.3897/mycokeys.138.186765

**Published:** 2026-08-06

**Authors:** Tai-Shun Li, Ying Zhang, Yan-Yan Yang, Hong-Bin Guan, Fang Wu, Qi Zhao

**Affiliations:** 1 State Key Laboratory of Efficient Production of Forest Resources, School of Ecology and Nature Conservation, Beijing Forestry University, Beijing 100083, China State Key Laboratory of Phytochemistry and Natural Medicines, Yunnan Key Laboratory for Fungal Diversity and Green Development, Kunming Institute of Botany, Chinese Academy of Sciences Kunming China; 2 State Key Laboratory of Phytochemistry and Natural Medicines, Yunnan Key Laboratory for Fungal Diversity and Green Development, Kunming Institute of Botany, Chinese Academy of Sciences, Kunming, Yunnan 650201, China Key Laboratory of Forest Disaster Warning and Control in Yunnan Province, Southwest Forestry University Kunming China; 3 Key Laboratory of Forest Disaster Warning and Control in Yunnan Province, Southwest Forestry University, Kunming, Yunnan 650224, China State Key Laboratory of Efficient Production of Forest Resources, School of Ecology and Nature Conservation, Beijing Forestry University Beijing China

**Keywords:** *
Basidiomycota
*, molecular data, morphology, new taxa, subgen. *Telamonia*

## Abstract

*Cortinarius* is renowned for its extraordinary species richness and cosmopolitan distribution. During a recent survey of macrofungal diversity across the Qinghai-Xizang Plateau, four specimens morphologically assignable to *Cortinarius* were collected. Based on detailed morphological characteristics and molecular phylogenetic analyses, these specimens are herein described as two new species within *Cortinarius* sect. *Telamonia*. *Cortinarius linzhiensis*, collected from mixed forests of *Quercus* and *Picea*, is characterized by a weakly hygrophanous, wrinkled pileus surface, a margin retaining spiderweb-like veil remnants, and a stipe apex occasionally exhibiting a pale violet tint. *Cortinarius pallidomarginatus*, collected from subtropical broadleaved forests dominated by *Castanopsis*, *Lithocarpus*, and *Cyclobalanopsis*, is characterized by its reddish-brown pileus with a contrasting white margin. This study provides detailed morphological descriptions, illustrations, and phylogenetic evidence supporting the recognition of these two novel taxa, along with comparisons with closely related species.

## Introduction

*Cortinarius* (Pers.) Gray, the largest agaricoid genus within the basidiomycete family *Cortinariaceae (Agaricales)*, spans nearly all forested biomes, from equatorial lowlands to subpolar tundra, attaining maximal alpha diversity in the cool-temperate zones of Europe, North America, and Asia (Kirk et al. 2008; Soop et al. 2019; Liimatainen et al. 2020, 2022). Species of *Cortinarius* are predominantly distributed in forest ecosystems, where they form ectomycorrhizal associations with a wide range of woody and herbaceous plants, playing crucial roles in ecosystem function and stability (Geml et al. 2012; Nouhra et al. 2013; Horton et al. 2017; Nuske et al. 2019).

Melot (1989) established *Cortinarius* sect. *Telamonia*, designating *C. torvus* (Fr.) Fr. as the type species. This section is traditionally characterized by a typically translucent pileus with a moist margin and a membranous context, as well as a stipe often exhibiting a violet tint (Melot 1989). With the description of additional taxa, the circumscription of the section has expanded, and its diagnostic features have been further refined (Niskanen 2014; Liimatainen et al. 2017; Naseer et al. 2020; Xie et al. 2020; Jia et al. 2025; Zhou et al. 2026). Currently, species within sect. *Telamonia* exhibit considerable morphological diversity, including pileus colors ranging from white-purple and yellowish-brown to purplish-brown, a hygrophanous surface covered with fibrous scales, a stipe surface with white fibers and a frequently purplish apex, veil colors varying from white to purple or brown, ellipsoid basidiospores with moderate verrucosity, and a duplex pileipellis with a prostrate epicutis.

In China, systematic research on the genus *Cortinarius* began relatively late and has historically lagged behind studies in Europe and North America. Nevertheless, recent advances in molecular techniques, together with intensified field surveys, have significantly advanced research on this genus and its allied genera in China. In recent years, numerous new species of *Cortinarius* have been described, highlighting the remarkable diversity of this group (Xie et al. 2020, 2021, 2022, 2025a, 2025b; Zhou et al. 2023; Fan et al. 2024; Long et al. 2024; Jia et al. 2025; Wang et al. 2025a; Wang et al. 2025b, 2025c; Yang et al. 2025; Xu et al. 2026; Li et al. 2026). During a recent survey of macrofungal diversity in the Yarlung Zangbo Grand Canyon National Nature Reserve in the Xizang Autonomous Region and border counties of Yunnan Province, four specimens assignable to *Cortinarius* were collected. Based on detailed morphological observations and molecular phylogenetic analyses, these specimens are described here as two new species within *Cortinarius* sect. *Telamonia*. Comprehensive descriptions, illustrations, and phylogenetic evidence are provided for these taxa.

## Materials and methods

### Sample collection

Four specimens morphologically corresponding to *Cortinarius* were collected from the Yarlung Zangbo Grand Canyon National Nature Reserve in the Xizang Autonomous Region and the border region of Yunnan Province, China, during July and August 2025. Habitat photographs were taken using a digital camera (Canon, Japan). Detailed information, such as collection time, location, geographic coordinates, altitude, and vegetation, was recorded. The specimens were processed promptly after collection, and some fresh fruiting bodies were dried using silica gel for use as molecular material, whereas the remaining specimens were dried in a food dryer at 40 °C for preservation. The newly collected specimens were deposited in the Herbarium of Cryptogams of the Kunming Institute of Botany, Chinese Academy of Sciences (**KUN-HKAS**), China.

### Morphological observation

Samples were examined using the method described by Zhao et al. (2015). Macroscopic and microscopic features were examined using a stereomicroscope (SteREO Discovery.V12, Carl Zeiss Microscopy GmbH, Germany), and microphotographs were taken using a compound microscope (Nikon ECLIPSE 80i, Nikon, Japan) fitted with a Nikon DS-Ri2 digital camera (Nikon, Japan). Color codes indicated in the descriptions were based on Kornerup and Wanscher (1981). Macroscopic characteristics included the shape, color, size, and surface features of the pileus; the color and thickness of the context; the color, mode of attachment, width, and density of the lamellae; and the color, shape, texture, and presence of a ring on the stipe. Microscopic characteristics included the shape, size, and surface ornamentation of the basidiospores; the shape and size of the basidia; the type and thickness of the pileipellis; and the presence of cystidia and clamp connections. Dried specimens were sectioned and mounted in a 5% KOH solution for microscopic observation. Congo red reagent was used for staining to facilitate observation of the microscopic structures. To observe the surface ornamentation of the basidiospores, hymenophoral fragments were cut from the dried specimens, mounted on aluminum stubs, coated with gold-palladium, and observed under a ZEISS Sigma 300 scanning electron microscope (SEM) at the Kunming Institute of Botany.

Microstructural measurements were performed using Image Frame Work v.0.9.7. Measurements were given as (*a*–)*b*–*c*(–*d*), where *a* denoted the minimum value, *d* the maximum value, and *b*–*c* the 90% confidence interval. The numbers of basidiospores (*n*), basidiomata (*m*), and specimens (*p*) measured were denoted as [*n*/*m*/*p*]. The *Q* value indicated the length-to-width ratio of the basidiospores, whereas mean *Q* indicated the average of the length-to-width ratios (*Q*) of all basidiospores ± standard deviation. The illustrations were prepared using Adobe Photoshop 2020 (Adobe Systems, USA).

### DNA extraction, PCR amplification, and sequencing

DNA was extracted directly from *Cortinarius* fruiting bodies using a Trelief™ Fungal Genomic DNA Extraction Kit (Tsingke Biotechnology Co., Ltd., Beijing, China). Polymerase chain reaction (PCR) was used to amplify the nuclear ribosomal internal transcribed spacer (ITS) region and the nuclear ribosomal large subunit (nrLSU) region. The ITS region was amplified using the universal primer pair ITS5/ITS4 (White et al. 1990). The nrLSU region was amplified using the universal primer pair LR0R/LR5 (Vilgalys and Hester 1990). The PCR conditions were as follows: an initial denaturation at 95 °C for 5 min; 35 cycles of denaturation at 95 °C for 30 s, annealing at 53 °C for 20 s, and extension at 72 °C for 30 s; and a final extension at 72 °C for 10 min. PCR products were sent to Tsingke Company in Beijing, China, for sequencing.

### Phylogenetic analyses

The forward and reverse sequences were assembled using DNASTAR Lasergene SeqMan Pro v.7.1.0. This study used newly generated sequence data and data from the literature to construct a phylogenetic tree (Table 1), primarily based on the research results of Liimatainen et al. (2020) and Zhou et al. (2026). Multiple sequence alignments were aligned using MAFFT v.7 (https://mafft.cbrc.jp/alignment/server/index.html, Katoh and Standley 2013; Katoh et al. 2019) and trimmed using TrimAl v.1.3 (Capella-Gutiérrez et al. 2009). The “gapthreshold” parameters for the ITS and nrLSU regions were set to 0.2 and 0.5, respectively. The trimmed sequences were assembled into a combined dataset in the order of ITS and nrLSU using SequenceMatrix v.1.8 (Vaidya et al. 2011).

**Table 1.** Names, voucher numbers, countries, references, and corresponding GenBank accession numbers of the taxa used in this study. Names shaded in blue indicate species newly described in this study. All type specimens are highlighted with a “**T**.” The symbol “–” denotes no available data.

**Species**	**Voucher**	**Locality**	**GenBank accession no**.		**References**
			**ITS**	**nrLSU**	
*Cortinarius agathosmus* **T**	CFP536	Sweden	KC608590	–	Niskanen et al. (2013)
*C. agathosmus*	H:T. Niskanen 10-005	Canada	MT934853	–	Liimatainen et al. (2020)
*C. agathosmus*	H:T. Niskanen 10-082	Canada	MT934852	–	Liimatainen et al. (2020)
*C. agathosmus*	H:I. Kytovuori 98-858	Sweden	MT934851	–	Liimatainen et al. (2020)
*C. alpigenus*	KUN-HKAS 97172	China	PX720763	–	Xu et al. (2026)
*C. alpigenus* **T**	KUN-HKAS 151734	China	PX720762	–	Xu et al. (2026)
*C. calopus* **T**	H P.A. Karsten 314a	Finland	NR_172325	–	Liimatainen et al. (2020)
*C. calopus*	LP_178	Canada	PQ436988	–	Unpublished
*C. calopus*	MICH:17713	USA	NR_170845	–	Liimatainen et al. (2020)
*C. calopus*	MM 1980-0389	USA	MT935167	–	Liimatainen et al. (2020)
*C. coriaceus* **T**	HKAS145316	China	PQ772202	PQ772214	Jia et al. (2025)
*C. fructuodorus* **T**	H 7001104	USA	NR_131827	–	Niskanen et al. (2013)
*C. fructuodorus*	H:T. Niskanen 12-308	USA	MT935066	–	Liimatainen et al. (2020)
*C. ionophyllus*	H:I. Kytovuori 97-137	Finland	MT935169	–	Liimatainen et al. (2020)
*C. ionophyllus*	MM 1949-0052	Austria	NR_172336	–	Liimatainen et al. (2020)
*C. leucophaeatus*	H:I. Kytovuori 97-138	Finland	MT935196	–	Liimatainen et al. (2020)
*C. linzhiensis* **T**	HKAS 153528	China	PZ072317	PZ072349	This study
*C. linzhiensis*	HKAS 153529	China	PZ072318	PZ072350	This study
*C. neotorvus* **T**	HMJAU44441	China	NR_182688	–	Xie et al. (2020)
*C. neotorvus*	HMJAU44437	China	MK552382	–	Xie et al. (2020)
*C. niveotraganus* **T**	H I. Kytovuori 98-033	Finland	NR_131842	–	Niskanen (2014)
*C. niveotraganus*	WTU:J.F. Ammirati 9927	USA	MT935253	–	Liimatainen et al. (2020)
*C. obscuratus*	KUN-HKAS 138920	China	PX720760	–	Xu et al. (2026)
*C. obscuratus* **T**	KUN-HKAS 138955	China	PX720759	–	Xu et al. (2026)
*C. odoritraganus* **T**	H 7057490	Canada	NR_170030	–	Niskanen (2020)
*C. odoritraganus*	MICH 10398	USA	NR_170852	–	Liimatainen et al. (2020)
*C. pallidomarginatus* **T**	HKAS 153526	China	PZ072319	PZ072351	This study
*C. pallidomarginatus*	HKAS 153527	China	PZ072320	PZ072352	This study
*C. pseudotorvus* **T**	LAH 35257	Pakistan	MN864285	–	Naseer et al. (2020)
*C. pseudotorvus*	CAL 1985	India	PP349829	PP342048	GenBank
*Cortinarius* sp.	WTU-F-073297	USA	MZ048728	–	GenBank
*C. subionophyllus*	TN 06-050	Sweden	MT935497	–	Liimatainen et al. (2020)
*C. subionophyllus*	LP_147	Canada	PQ437004	–	GenBank
*C. subpulchrifolius* **T**	MICH 10419	USA	NR_170855	–	Liimatainen et al. (2020)
*C. subpulchrifolius*	H:19.07.2009	Canada	MT935517	–	Liimatainen et al. (2020)
*C. tigrinipes* **T**	G 874	France	NR_171371	–	Liimatainen et al. (2020)
*C. tigrinipes*	H:T. Niskanen 04-1120	Italy	MT935550	–	Liimatainen et al. (2020)
*C. torvus*	ACAD 21403F	Canada	PV248385	–	Unpublished
*C. torvus*	PBM4036	USA	MG663302	MF797696	Unpublished
*C. torvus*	PBM4035	USA	MG663305	MF797704	Unpublished
*C. torvus*	PV_113	Belgium	PX221210	–	Unpublished
*C. torvus* **T**	CFP778	Sweden	MT935556	–	Liimatainen et al. (2020)
*C. torvus*	T35	Norway	KC842400	KC842471	Stensrud et al. (2014)
*C. traganus* **T**	S H. Lindstrom CFP763	Sweden	NR_172341	–	Liimatainen et al. (2020)
*C. traganus*	KH10	Norway	KC842399	KC842470	Stensrud et al. (2014)
*C. traganus* **T**	WTU J.F. Ammirati 9578	USA	NR_172352	–	Liimatainen et al. (2020)
*C. ultimiionophyllus*	H:I. Kytovuori 95-507	Finland	MT935569	–	Liimatainen et al. (2020)
*C. ultimiionophyllus*	H:T. Niskanen 07-303	Canada	MF379635	–	Unpublished
*C. venustissimus* **T**	PC 5371	Sweden	NR_171163	–	Liimatainen et al. (2020)
*C. venustissimus*	H:T. Niskanen 03-938	Sweden	MT935584	–	Liimatainen et al. (2020)
*C. venustus*	F16351	Canada	FJ039572	FJ039572	Harrower et al. (2011)
*C. venustus* **T**	H:P.A. Karsten 3234	Finland	MT935132	–	Liimatainen et al. (2020)

Maximum likelihood (ML) analysis was performed on the CIPRES Science Gateway platform (http://www.phylo.org/) using RAxML-HPC2 v.8.2.12 (Miller et al. 2010). The GTR+I+G substitution model was used for the ML analysis of all gene regions, with 1,000 rapid bootstrap replicates. Bayesian inference (BI) was performed using MrBayes v.3.2 with Markov chain Monte Carlo (MCMC) sampling to calculate posterior probabilities (PP). The best-fit evolutionary model for each gene region was determined using MrModeltest v.2.3 based on the Akaike information criterion (Nylander et al. 2004). The GTR+G and HKY+I substitution models were selected for the ITS and nrLSU regions, respectively. Four Markov chains were run simultaneously for 305,000 generations, with sampling every 100 generations. The first 25% of trees were discarded as burn-in, and convergence was considered achieved when the standard deviation of split frequencies fell below 0.01 and the effective sample size (ESS) exceeded 200. The phylogenetic tree was visualized using FigTree v.1.4.4 and edited using Adobe Illustrator CC 2018 (Adobe Systems, USA).

## Results

### Molecular analyses

**Figure 1.**
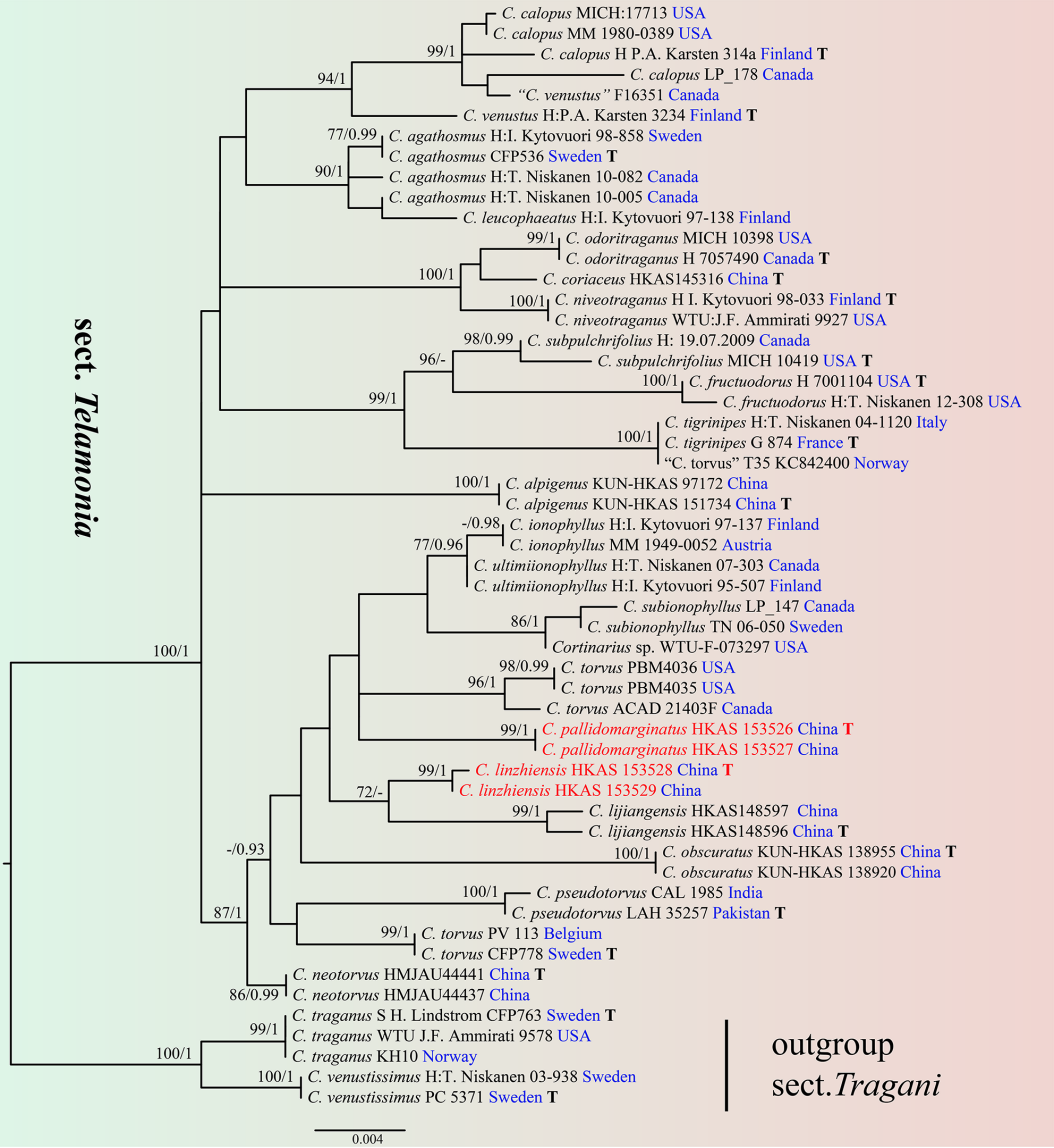
Maximum likelihood phylogenetic tree generated from the combined ITS and nrLSU sequence dataset. Maximum likelihood bootstrap support (MLBS) ≥ 70% and Bayesian inference posterior probability (BIPP) ≥ 0.90 are indicated above the nodes. “-” indicates that the support value was below the respective threshold. Specimen vouchers and countries are indicated after the species names. New samples collected in this study are indicated in red. “T” indicates that the type specimens.

This dataset contained a total of 64 sequences, including eight newly generated sequences and 56 downloaded sequences (54 ITS and 10 nrLSU), representing 22 species. The alignment contained 1,555 characters, including gaps (ITS: 1–639; nrLSU: 640–1,547). The two new species clustered within sect. *Telamonia*. Following Liimatainen et al. (2020), the closest section, sect. *Tragani*, was selected as the outgroup. The ML and BI analyses showed similar topologies, and the topology obtained from the ML analysis is presented with the statistical support values from the MLBS and BIPP analyses (Fig. 1).

### Taxonomy

#### Cortinarius linzhiensis

Taxon classificationFungiAgaricalesCortinariaceae

T.S. Li, F. Wu & Q. Zhao sp. nov.

4806D6A3-A4FB-5F33-985C-A83C2030B7C6

862688

Fig. 2

##### Chinese name.

林芝丝膜菌 (lin zhi si mo jun).

**Figure 2.**
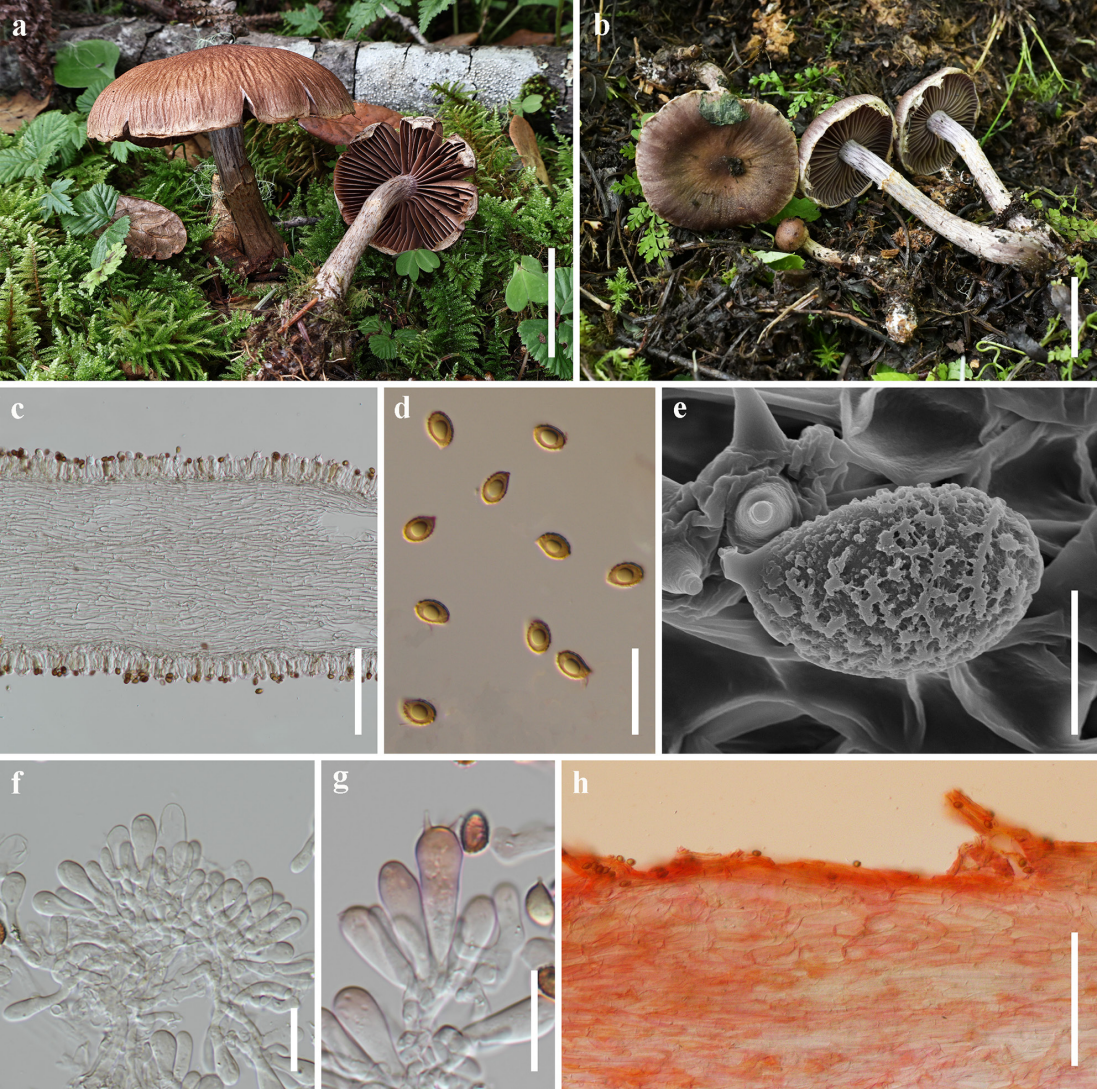
*Cortinarius linzhiensis*. **a, b**. Basidiomata (**a**. HKAS 153528, holotype; **b**. HKAS 153529); **c**. Lamellar trama; **d**. Basidiospores; **e**. Scanning electron micrograph of basidiospores; **f**. Marginal elements; **g**. Basidia; **h**. Pileipellis. Scale bars: 3 cm (**a, b**); 3 μm (**e**); 20 μm (**d, f**); 100 μm (**c, h**).

##### Etymology.

“linzhiensis” refers to the type specimen collected from Linzhi City.

##### Holotype.

China • Xizang Autonomous Region, Linzhi City, Bayi District, Lulang Town, Gongcuo Lake Scenic Area, at 29.764327°N, 94.753644°E, alt. 3420 m, ground in mixed forests of *Quercus aquifolioides* and *Picea likiangensis* var. linzhiensis, 4 Aug. 2025, LTS657 = HKAS 153528.

##### Diagnosis.

*Cortinarius linzhiensis* resembles *C. lijiangensis* Y. Zhou, Y. Zhang & Q. Zhao, but differs in having a brown to dark brown pileus and thinner epicutis (Zhou et al. 2026).

##### Macromorphology.

***Basidiomata*** medium-sized. ***Pileus*** 40–90 mm diam., initially slightly hemispherical to campanulate, becoming applanate to umbonate with age; surface weakly hygrophanous, wrinkled, light brown to brown (7D5–7D8), reddish brown (8E4–8E6), to dark brown (9F4–9F5), gradually deepens towards the center, densely covered with fibrillose squamules; margin cracked up to halfway up the pileus, with grayish-white spiderweb-like remnants of the veil, turning brown upon maturity; context 2–7 mm thick, white. ***Lamellae*** emarginate, 6–14 mm broad, moderately crowded, dark reddish brown to dark brown (8F7, 8F8–9E4). ***Stipe*** 70–165 × 7–25 mm, cylindrical, usually tapering upwards; surface dirty white, sometimes with a pale purple (17E6) tinge at the apex, covered with brown fibrillose squamules; leaving a pale yellow (5C5) ring on the upper part of the stipe. Basal mycelium white. ***Odor*** not distinctive.

##### Micromorphology.

***Basidiospores*** [100/2/2] (8–) 8.5–10 (–11) × 5.5–7 (–7.5) μm [*Q* = (1.28) 1.32–1.65 (–1.84), *Q* = 1.47 ± 0.08], ellipsoid to amygdaliform, cinnamon in KOH, moderately verrucose. ***Basidia*** 26–37 × 7–10 μm, clavate, 4-spored. ***Lamellae trama*** hyphae 5–23 μm diam., smooth, colourless to yellowish. ***Cystidia*** absent. Marginal elements (*n* = 20) 23–34 × 8–10 μm, mostly inflated, clavate, apex round. ***Pileipellis*** duplex: epicutis weakly developed, 15–45 μm thick, composed of interwoven to parallel, colorless, smooth, thin-walled, long-celled hyphae 4.5–8 μm diam.; hypocutis composed of interwoven to parallel, cylindrical, colourless, thin-walled hyphae 10–22 μm diam. ***Stipe hyphae*** 5–10 μm diam., colourless, smooth. ***Clamp connections*** present.

##### Habitat.

Mycorrhizal and gregarious in coniferous forests dominated by trees of *Picea* spp. and *Quercus* spp.

##### Distribution.

Known from the southeastern part of the Xizang Autonomous Region, China.

##### Additional material examined.

China • Xizang Autonomous Region, Linzhi City, Bayi District, Lulang Town, at 29.657897°N, 94.723772°E, alt. 3,815 m, ground in the *Picea* forests, 5 Aug. 2025, GuHB-137 = HKAS 153529.

##### Notes.

*Cortinarius linzhiensis* is characterized by a light brown to dark brown pileus with a wrinkled surface, dark reddish brown to dark brown lamellae, a dirty white stipe, and ellipsoid to amygdaliform basidiospores. Phylogenetic analysis indicates that *C. linzhiensis* is sister to *C. lijiangensis* (Fig. 1), although with moderate bootstrap support. *Cortinarius lijiangensis* is distributed in subalpine *Picea*–*Abies* forests in southwestern China and exhibits habitat preferences similar to those of *C. linzhiensis*. Morphologically, *Cortinarius linzhiensis* differs from *C. lijiangensis* in having a brown, reddish brown to dark brown pileus, a dirty white stipe with a pale yellow annular zone, and a thinner epicutis (15–45 μm). In contrast, *C. lijiangensis* possesses a maroon pileus with a grayish-yellow margin, a maroon stipe, an evanescent arachnoid annulus, and a significantly thicker epicutis (140–230 μm) (Zhou et al. 2026).

#### Cortinarius pallidomarginatus

Taxon classificationFungiAgaricalesCortinariaceae

T.S. Li, F. Wu & Q. Zhao sp. nov.

45C96E4A-43E9-56A3-B636-C6DD81F1FA13

862689

Fig. 3

##### Chinese name.

淡缘丝膜菌 (dan yuan si mo jun).

**Figure 3.**
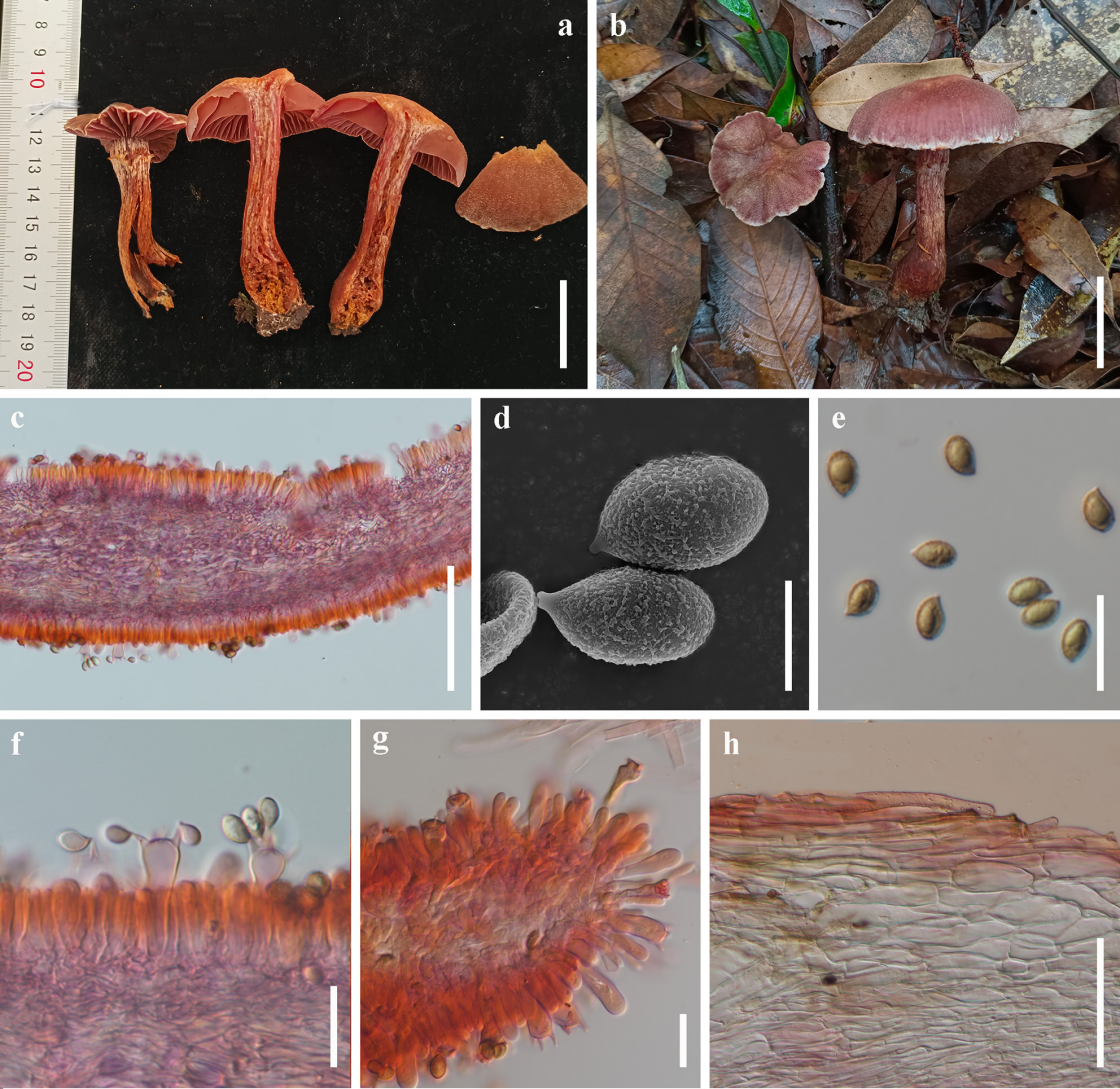
*Cortinarius pallidomarginatus*. **a, b**. Basidiomata (**a, b**. HKAS 153526, holotype); **c**. Lamellar trama; **d**. Scanning electron micrograph of a basidiospore; **e**. Basidiospores; **f**. Basidia; **g**. Marginal elements; **h**. Pileipellis. Scale bars: 3 cm (**a, b**); 5 μm (**d**); 20 μm (**e–g**); 100 μm (**c, h**).

##### Etymology.

“pallidomarginatus” refers to the reddish-brown pileus with a white margin.

##### Holotype.

China • Yunnan Province, Tengchong City, Houqiao Town, at 25.1610°N, 98.1848°E, alt. 1770 m, ground in the *Castanopsis*, *Lithocarpus*, and *Cyclobalanopsis* forests, 31 Jul. 2025, LTS861 = HKAS 153526.

##### Diagnosis.

*Cortinarius pallidomarginatus* resembles *C. subionophyllus* Niskanen, Liimat. & Kytov. and *C. ultionophyllus* Kytov., Niskanen & Liimat., but differs in having a reddish-brown pileus and lamellae (Liimatainen et al. 2017).

##### Macromorphology.

***Basidioma*** medium-sized. ***Pileus*** 40–60 mm diam., initially slightly campanulate, becoming applanate to umbonate with age, sometimes with an uneven surface; surface weakly hygrophanous, reddish brown (8E5–8E8), densely covered with fibrillose squamules, margin white (1A1), with reddish brown spiderweb-like remnants of the veil; context 2–6 mm thick, light reddish brown (8D4). ***Lamellae*** emarginate, 3–9 mm broad, moderately crowded, reddish brown (8E6). ***Stipe*** 60–80 × 9–17 mm, cylindrical, slightly curved, gradually widening towards the base; surface light reddish brown (9D4), covered with light brown fibrillose squamules; leaving a pale yellow (5C5) ring on the upper part of the stipe. Basal mycelium white. ***Odor*** not distinctive.

##### Micromorphology.

***Basidiospores*** [70/2/2] (8–) 8.5–10 × (5.5–) 6–7 μm [*Q* = (1.29–) 1.31–1.56 (–1.58), *Q* = 1.43 ± 0.06], ellipsoid to amygdaliform, brown in KOH, moderately verrucose. ***Basidia*** 23–45 × 8.5–11 μm, clavate, 4-spored. ***Lamellae trama*** hyphae 5–18 μm diam., smooth, colourless to yellowish. ***Cystidia*** absent. Marginal elements (*n* = 20) 26–36 × 7–9 μm, mostly inflated, clavate, apex round. ***Pileipellis*** duplex: epicutis weakly developed, 40–75 μm thick, composed of interwoven to parallel, colorless, smooth, thin-walled, long-celled hyphae 6.5–13 μm diam.; hypocutis composed of interwoven to parallel, cylindrical, colourless, thin-walled hyphae 13–27 μm diam. ***Stipe hyphae*** 6–25 μm diam., colourless, smooth. ***Clamp connections*** present.

##### Habitat.

Mycorrhizal, gregarious in broad-leaved forests dominated by *Castanopsis*, *Lithocarpus*, and *Cyclobalanopsis* trees.

##### Distribution.

Known from the southwestern Yunnan Province, China.

##### Additional material examined.

China • Yunnan Province: Tengchong City, Houqiao Town, at 25.1610°N, 98.1848°E, alt. 1770 m, ground in the *Castanopsis*, *Lithocarpus*, and *Cyclobalanopsis* forests, 31 Jul. 2025, LTS861-1 (HKAS 153527).

##### Notes.

*Cortinarius pallidomarginatus* is characterized by its reddish-brown pileus with a white margin, reddish-brown lamellae, light reddish-brown stipe, and ellipsoid to amygdaliform basidiospores. Phylogenetic analysis indicates that *C. pallidomarginatus* forms a sister clade with a misidentified *C. torvus*-like clade, albeit with low bootstrap support (Fig. 1). Additionally, the new species is phylogenetically closely related to *Cortinarius subionophyllus* and *C. ultionophyllus*. Morphologically, *Cortinarius pallidomarginatus* can be readily distinguished from *C. ultimiionophyllus* and *C. subionophyllus* by its reddish-brown pileus with a distinct white margin. In contrast, both *C. ultimiionophyllus* and *C. subionophyllus* have a grayish-purplish-brown to ochraceous-brown pileus without a white margin. Ecologically, *Cortinarius pallidomarginatus* is restricted to subtropical broadleaved forests in East Asia, whereas the latter two species are distributed in the boreal forests of Northern Europe and North America (Liimatainen et al. 2017).

## Discussion

Based on morphological characteristics and phylogenetic analysis of the ITS + nrLSU dataset, this study describes two new species of *Cortinarius* sect. *Telamonia*, namely, *C. linzhiensis* and *C. pallidomarginatus*. In the phylogenetic framework, both species are placed within sect. *Telamonia*, albeit with relatively low bootstrap support. Nevertheless, they exhibit several macroscopic and microscopic features consistent with this section, supporting their taxonomic placement within the section.

Both species share key diagnostic characters, including a weakly hygrophanous, densely fibrous-scaly pileus, a fibrous-scaly stipe, and a white to dirty-white veil that gradually forms a distinct ring as it matures. Microscopically, they possess ellipsoid to amygdaliform basidiospores with moderately verrucose ornamentation and a duplex pileipellis with appressed hyphae in the epicutis, all of which are typical of sect. *Telamonia*. Notably, *C. pallidomarginatus* exhibits a reddish-brown pileus, a relatively uncommon feature in sect. *Telamonia*, in which white, yellowish-brown, and purplish-brown colors predominate.

Both specimens of *Cortinarius linzhiensis* were collected from Lulang Town, Linzhi City, Xizang Autonomous Region: one from a mixed forest dominated by *Quercus* and *Picea* at an altitude of 3,420 m (HKAS 153528), and the other from a *Picea*-dominated coniferous forest at an altitude of 3,815 m (HKAS 153529). In the phylogenetic analysis, these two specimens clustered together, forming an independent lineage within sect. *Telamonia* (Fig. 1). It should be emphasized that the two collections differ by two nucleotide substitutions and insertion–deletion (indel) sites in the ITS sequences, as well as in pileus color and pigmentation of the stipe apex. Such subtle phenotypic differences represent intraspecific morphological variation caused by differences in altitude and host forest environments. These variable color traits are not considered diagnostic for interspecific delimitation in this study, and the two collections are therefore confidently recognized as conspecific specimens of *Cortinarius linzhiensis*.

Sect. *Telamonia* is an important section within *Cortinarius* subgen. *Telamonia*, and the phylogenetic positions of its constituent species are relatively stable. In recent years, many new species have been reported within this section (Liimatainen et al. 2017; Naseer et al. 2020; Xie et al. 2020; Jia et al. 2025; Xu et al. 2026; Zhou et al. 2026). However, some taxonomic issues remain unresolved. Sequences identified as *C. torvus* form two distinct clades: one clusters with the neotype, whereas the other is sister to the newly described species *C. pallidomarginatus*, suggesting that this lineage may represent a potential new species. In the phylogenetic analysis, sequence KC842400, identified as *C. torvus*, clusters with *C. tigrinipes* Bergeron (Fig. 1), indicating a likely misidentification and potential misapplication of the name *C. torvus*.

Three core protein-coding loci (RPB1, RPB2, and TEF1-α) were sequenced to strengthen the molecular evidence and improve species delimitation. However, most published reference species of sect. *Telamonia* in public databases lack these high-resolution markers. The absence of protein-coding sequence data results in fragmented multilocus alignments and unstable phylogenetic topologies. Consequently, the ITS + nrLSU dataset was selected for the primary phylogenetic reconstruction to maximize taxon coverage. The newly obtained multilocus sequences provide supplementary evidence supporting the independent evolutionary status of the two new species.

## Supplementary Material

XML Treatment for Cortinarius linzhiensis

XML Treatment for Cortinarius pallidomarginatus

## Additional information

### Artificial Intelligence (AI) use

The authors accept full responsibility for the content of the manuscript, including the disclosure of any use of AI.

No AI tools were used in the preparation of this manuscript.

### Author ORCIDs

T.-S. Li https://orcid.org/0000-0001-8697-1630

Y. Zhang https://orcid.org/0009-0000-5248-0171

Y.-Y. Yang https://orcid.org/0009-0007-9521-558X

H.-B. Guan https://orcid.org/0009-0007-4113-2615

F. Wu https://orcid.org/0000-0002-1455-6486

Q. Zhao https://orcid.org/0000-0001-8169-0573

### Data availability

All of the data that support the findings of this study are available in the main text.
